# Skeletal Phenotype in Mulibrey Nanism, A Monogenic Skeletal Dysplasia With Fibrous Dysplasia

**DOI:** 10.1111/cge.14647

**Published:** 2024-11-18

**Authors:** Susann Karlberg, Sanna Toiviainen‐Salo, Marita Lipsanen‐Nyman, Outi Mäkitie

**Affiliations:** ^1^ Children's Hospital, Pediatric Research Center University of Helsinki and Helsinki University Hospital Helsinki Finland; ^2^ Folkhälsan Research Center Helsinki Finland; ^3^ Research Program for Clinical and Molecular Metabolism, Faculty of Medicine University of Helsinki Helsinki Finland; ^4^ Department of Pediatric Radiology HUS Diagnostic Center, University of Helsinki and Helsinki University Hospital Helsinki Finland; ^5^ Department of Molecular Medicine and Surgery Karolinska Institutet and Clinical Genetics, Karolinska University Hospital Stockholm Sweden

**Keywords:** fibrous dysplasia, MUL, Mulibrey nanism, skeletal dysplasia, TRIM37

## Abstract

Mulibrey nanism (MUL) is a monogenic growth disorder caused by mutations in *TRIM37*, with pre‐and postnatal growth failure, typical craniofacial features, perimyocardial heart disease, infertility and predisposition to tumors. Clinically, patients are gracile with relative macrocephaly, thin extremities, and narrow shoulders, but the full spectrum of skeletal features remains unknown. We conducted a cross‐sectional study in order to further clarify the skeletal phenotype. We assessed radiographs of the long bones and spine in 33 MUL patients, aged 4.5–48 years (14 females and 19 males, median age 16.7 years) for skeletal features. Hospital records were reviewed for clinical characteristics and fractures. Results confirmed significant skeletal abnormalities related to MUL. Skeletal changes were present in all patients; long bones were slender and bowed with broad metaphyses and narrow diaphysis, the cortices were thick, and medullary cavities were narrow. The vertebral bodies were tall. Fibrous dysplasia was found in 19/33 patients (58%); changes were monostotic in 58% and polyostotic in 42%. Altogether 17/33 patients (52%) had a history of fractures. This study confirms that in addition to short stature, patients with MUL have a specific skeletal dysplasia. Our findings suggest an important role for TRIM37 in cellular functions governing skeletal modelling and remodelling.

## Graphical abstract

We present a cross‐sectional detailed radiographic evaluation of the skeletal phenotype in 33 patients, aged 4.5–48 years, with Mulibrey nanism (MUL). This study confirms MUL as a skeletal dysplasia with prenatal‐onset growth failure, slender bones, vertebral changes, and a high prevalence of fibrous dysplasia and fractures.

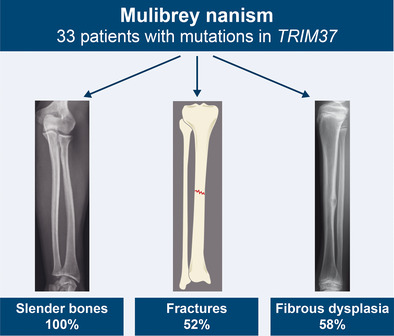

## 1 Introduction

Mulibrey nanism (MUL; OMIM **#**253250) is an autosomal recessive disorder with pre‐ and postnatal growth failure, typical craniofacial features, perimyocardial heart disease, hypogonadism, infertility, and predisposition to tumors [1, 2, 3, 4, 5]. MUL is caused by truncating mutations in the *TRIM37* gene, encoding TRIM37 protein with ubiquitin E3 ligase activity [6, 7, 8]. The disease pathogenesis is still unknown, but increasing evidence links MUL to defects in centrosome biology [9]. Worldwide, approximately 150 patients with MUL have a genetically confirmed diagnosis, some 110 of whom are Finnish. The Finnish patients are genotypically homogenous, over 90% being homozygous for the Finnish founder mutation (Fin‐major, c. 493‐2A>G). For decades, the clinical care and follow‐up of Finnish MUL patients were conducted at our institution, thereby enabling accumulation of unique expertise on this rare patient group.

Most patients (95%) are born small for gestational age (average birth length −3.1 SDS and birth weight −2.8 SDS). Postnatal growth shows no catch‐up and growth failure progresses [1]. The median adult height for women is 136 cm and for men, 150 cm [1].

Clinically, patients with MUL are gracile and have relative macrocephaly, thin extremities with proximal shortness and narrow shoulders. The characteristic facial features include triangular face with high, broad forehead and low nasal bridge. Skull radiographs show occipitofrontal bossing (in 90% of patients), low and shallow (J shaped) sella turcica (89%), laterally rotated orbital fossae (80%), small hypoplastic face (71%) and orbital hypertelorism (64%) [1].

General radiological findings have previously been analyzed at the time of diagnosis, at median age of 2.1 years, in a study from 2004. In this analysis, most patients presented with slender long bones with thick cortex and narrow medullary channel along with bell‐shaped thorax and thin ribs; 25% of the patients had fibrous dysplasia‐like lesions of long bones. In addition, 20% had a slight asymmetry of extremities [1]. However, the full spectrum of skeletal features remains unknown. In this work, we conducted a retrospective cross‐sectional study to further clarify the skeletal phenotype and possible age‐related changes.

## 2 Material and Methods

### 2.1 Patients

The study cohort included 33 patients (19 females, 14 males), aged 4.5–48 years at the time of radiographic examination (17 children or adolescents, and 16 adults; median age 16.7 years), who had been followed at the Children's Hospital and had a full skeletal survey available for analysis. The cohort was unselected regarding the severity of the disease. All had a genetically confirmed diagnosis; 32 patients were homozygous for the Fin‐major mutation (*TRIM37* c. 493‐2A>G) and one, compound heterozygous for the Fin‐major and the Fin‐minor mutations (c.2212delG). Hospital records were retrospectively analyzed for clinical characteristics, growth hormone (GH) treatment and fractures. Fractures were categorized into low, moderate or high energy traumas. All patients and/or their guardians provided an informed written consent.

### 2.2 Radiographic Analyses

All accessible radiographs of the long bones and spine were assessed for skeletal features by a radiologist with expertise on skeletal dysplasia (S.T.‐S.). At least 12 skeletal radiographs for each patient were available for review.

## 3 Results

### 3.1 Clinical Characteristics

All patients were born small for gestational age with a median birth length of 44 cm (−3.5 SDS) and weight of 2205 g. Median adult height was 152 cm (127–164 cm) for males and 142 cm (132–156 cm) for females. Of the 33 patients, 28 (85%) had received or were receiving recombinant human GH treatment. Median age for initiation of GH treatment was 4.8 years. The median treatment duration in patients who had completed GH therapy was 10.0 years.

### 3.2 Radiographic Findings

Radiographic analysis confirmed significant skeletal abnormalities related to MUL. Skeletal changes were present in all patients.

#### 3.2.1 Long Bones

**FIGURE 1:**
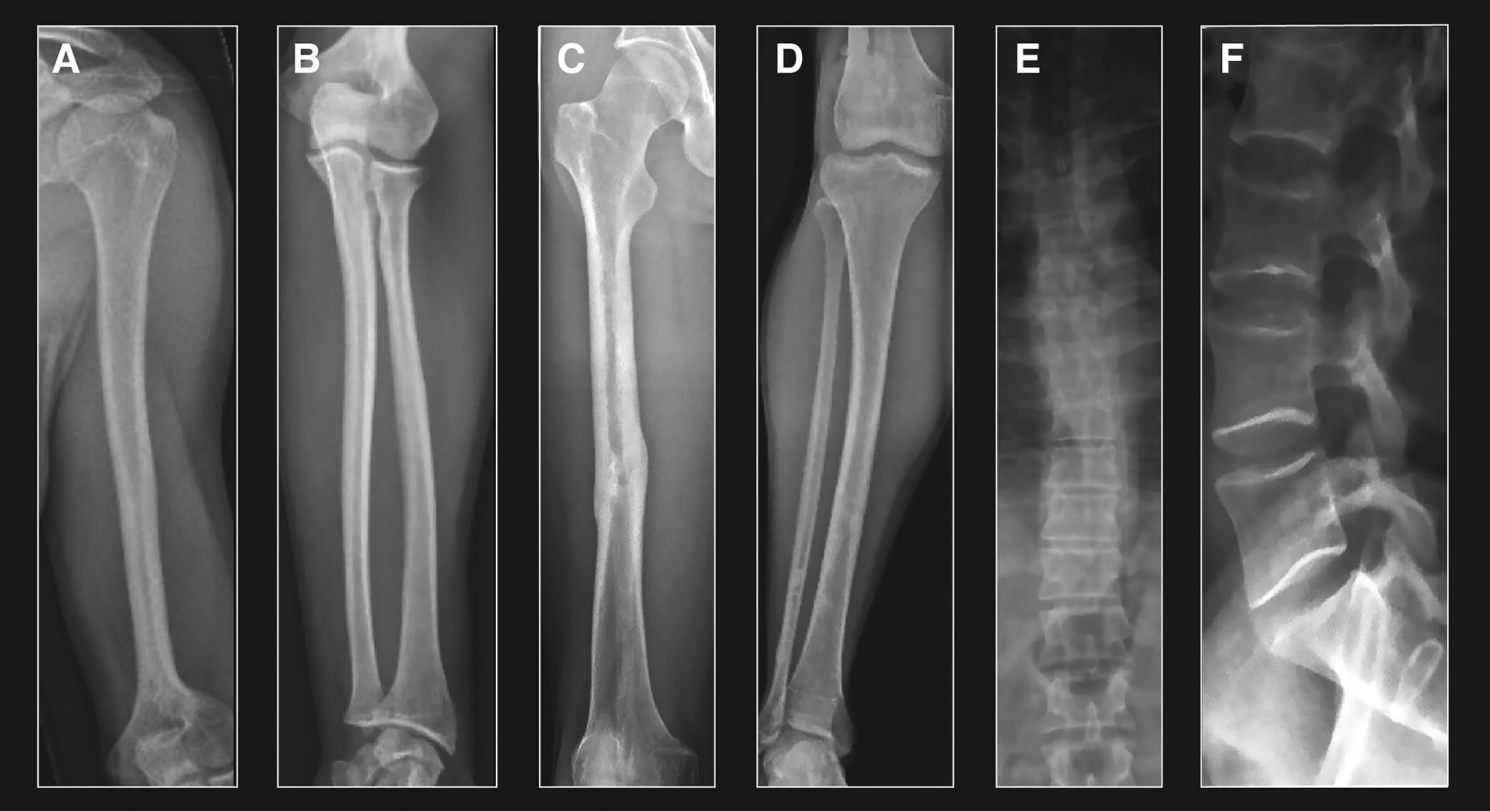
Skeletal features in a 39 years old male patient. The long bones are slender, cortices thick and medullar cavities narrow. (A) In the humerus, cortical thickness is most pronounced distally. Note also the especially broad metaphysis. (B) In the radius and ulna, cortical thickness is most prominent centrally. Note also the slight bowing of the bones. (C) In the femur, cortical thickness is most pronounced proximally. Note the old fracture in mid‐shaft with impaired healing. (D) In the tibia and fibula, cortical thickness is most prominent centrally. Note the areas of irregular cortex indicative of FD in the distal half of both the tibia and fibula. (E) The spine shows signs of mild scoliosis but normal vertebral width. (F) Vertebral bodies are tall with concave endplates. This patient had no history of GH therapy.

The long bones were slender and gracile in all 33 patients. The metaphyses were broad in comparison with the narrow diaphysis suggesting overmodelling. In those with open growth plates the secondary ossification centers appeared normal in shape and size. The femoral necks were in valgus position. The long bones had thick cortices and narrow medullary cavities (Figure 1). Cortical thickness was most pronounced in the femur and radius. The location of the cortical thickness varied. In the femur, cortical thickness was located proximally, in the humerus distally and in the tibia, fibula, radius and ulna centrally (Figure 1A–D). Slight bowing of long bones was observed in all patients. Bowing of the radius was most frequent, observed in 31/33 (94%) of patients (Figure 1B), followed by bowing of the fibula in 25/33 patients (76%). Bowing of the femur was noted only in 7/33 patients (21%). None of the patients had required osteotomy for correction of bowing deformity. A slight asymmetry of the lower limb length was noted in 7/33 patients (21%).

#### 3.2.2 Spine

**FIGURE 2:**
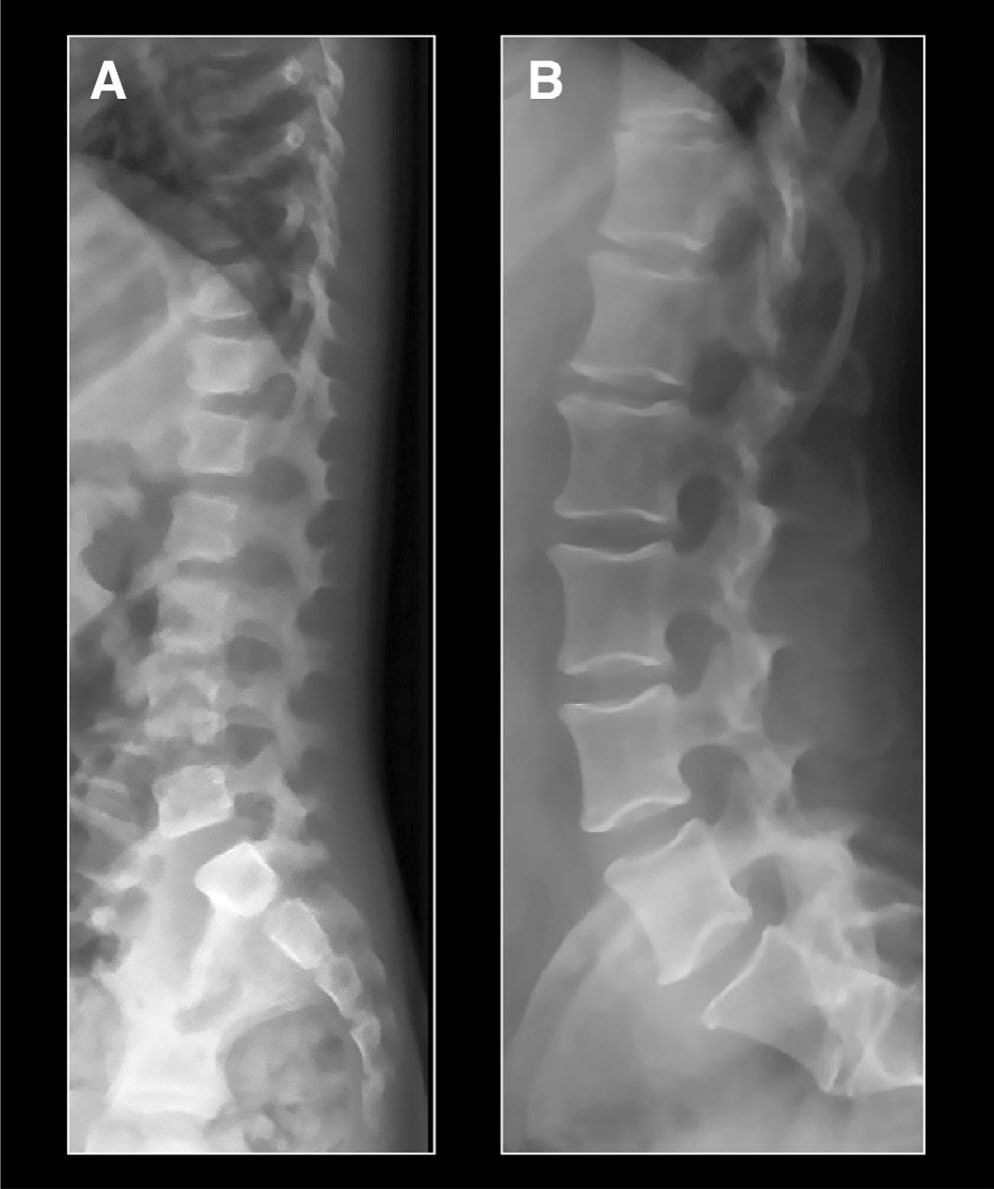
(A) Vertebral features in a 5 years old patient and (B) in a 48 years old patient. Both patients have relatively tall vertebrae but no other vertebral abnormalities. Note the especially tall vertebral bodies and the concave endplates in the lumbar area of the adult patient.

The vertebral bodies were tall in all patients who had attained adult height, with an increased ratio between the vertical diameter and the sagittal diameter (Figures 1E,F and 2B). This was especially apparent in the lumbar area. Vertebral endplates were concave. No major spinal abnormalities, including vertebral anomalies, were observed but seven patients had radiological signs of mild scoliosis that had not needed bracing or surgery. None of the patients had signs of compression fractures. Already in growing children, the vertebral bodies seemed tall (Figure 2A). The vertebral features were similar in patients with and without GH treatment.

#### 3.2.3 Fibrous Dysplasia

**FIGURE 3:**
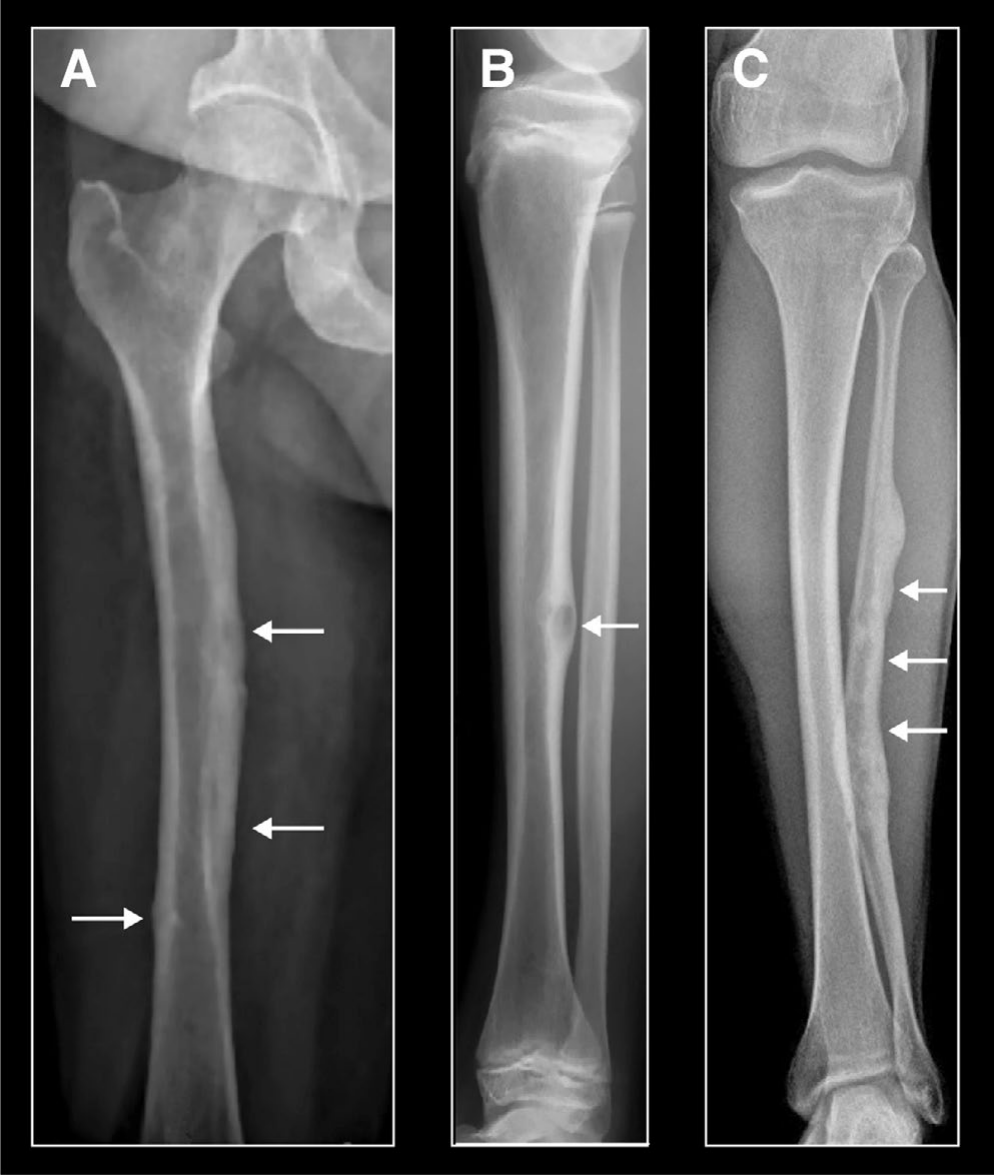
(A) Fibrous dysplasia of the femur in a 48 years old woman, (B) of the tibia in a 13 years old boy and (C) of an extended area of the fibula in a 39 years old male. Note also the deformity of the fibula as a result of a previous fracture in the FD weakened bone (C). Arrows indicate areas of FD.

Radiographic changes corresponding to FD were found in 19/33 patients (58%); changes were monostotic in 58% and polyostotic in 42%. FD was equally frequent in females and males (58% and 57%, respectively). In children, FD was found in 4/6 boys (67%) and 7/11 girls (64%). In adults, FD was observed in 50%. FD lesions were encountered in all long bones but were most often present in the tibia (9/19 patients, 47%) (Figure 3). No lesions were observed in the spine, pelvis, hands or feet, or other bones outside the long bones.

#### 3.2.4 Fractures

Altogether 17/33 patients (52%) had a history of fractures, 8/14 males (57%) and 9/19 females (47%). The majority of patients, 65%, sustained their first fracture before 10 years of age (Table 1). Half of those with a positive fracture history (9/17, 53%) had experienced more than one fracture. Most fractures were located in the radius or tibia. The mechanisms of injury were mostly falls during for instance play or exercise; high‐energy fractures were uncommon (Table 1). FD was observed in 65% of patients with fractures. In 45% of these patients, the fractures occurred in an area where FD was radiologically suspected or had been diagnosed earlier (Table 1). All patients with polyostotic FD had a history of at least one fracture. Of the children, 65% (11/17) had sustained fractures.

**TABLE 1:** Characterization of 1. fracture in 17 patients with Mulibrey nanism.

Nr	M/F	Age^a^	Localization	Trauma^b^	Subsequent fractures (*n*)	FD^c^	FD suspicion at fracture site	GH at time of fracture^d^	GH before fracture
1	F	0–5	Humerus	Low energy	1	Yes	No	No	No
2	F	0–5	Radius	Low energy	1	No	—	No	No
3	F	0–5	Radius	Low energy	2	No	—	Yes	Yes
4	F	0–5	Skull	Moderate energy	0	Yes	No	Yes	Yes
5	M	0–5	Finger	Low energy	1	Yes	No	Yes	Yes
6	M	0–5	Tibia	Low energy	7	Yes	Yes	No	No
7	F	5–10	Radius	Low energy	1	No	—	Yes	Yes
8	M	5–10	Tibia	Moderate energy	0	Yes	Yes	Yes	Yes
9	M	5–10	Radius	Low energy	0	Yes	No	Yes	Yes
10	M	5–10	Radius	Low energy	0	Yes	Yes	Yes	Yes
11	M	5–10	Tibia	Moderate energy	2	Yes	Yes	No	No
12	F	10–16	Radius	Moderate energy	0	Yes	No	Yes	Yes
13	F	10–16	Humerus	Moderate energy	0	Yes	No	Yes	Yes
14	M	10–16	Tibia	Moderate energy	1	No	—	Yes	Yes
15	F	10–16	Clavicula	High energy	0	No	—	No	Yes
16	M	10–16	Radius	Low energy	0	No	—	Yes	Yes
17	F	> 16	Fibula	High energy	1	Yes	Yes	No	Yes

^a^ Age group at first fracture in years.

^b^ Low energy trauma: fall from for example standing, play or light exercise, moderate energy: fall from < 3 m or equivalent, high energy: motorized vehicle accident or fall from > 3 m.

^c^ FD, Fibrous dysplasia.

^d^ GH, Growth hormone.

#### 3.2.5 Correlation With Clinical Features

No gender differences in the skeletal phenotype were observed. There was also no apparent clinical correlation between the degree of skeletal abnormalities and the overall disease severity. Further, the characteristic changes were not age dependent. The long bone cortices were similarly thick and medullary cavities narrow at all ages. Also, slight long bone bowing was evident already in childhood and the degree of bowing did not appear to be age‐related. The vertebrae were tall in all patients with completed growth but appeared somewhat tall already in childhood. The skeletal findings of the four patients without GH treatment did not considerably differ from the other patients; two of them also had a history of fractures. The degree of skeletal dysplasia varied despite identical genotype in most of the patients. The skeletal phenotype of the compound heterozygous patient did not significantly differ from the other patients. At the age of 4.5 years, the patient presented with slender long bones with thick cortices, slight bowing of long bones and FD in the tibia. The patient had not experienced any fractures thus far. Altogether 15/17 patients with GH treatment had sustained fractures and of these, 11/15 (73%) had started GH treatment before the first fracture (Table 1). There was no substantial difference in risk factors between the patients with and without fractures, apart from a slightly higher prevalence of FD in the fracture group (Table 2).

**TABLE 2:** Potential risk factors during childhood contributing to fractures in patients with and without fractures in the cohort of 33 subjects with Mulibrey nanism.

Characteristic	Fractures	No fractures
Number of patients, *n* (%)	17/33 (52)	16/33 (48)
Age at radiological examination (median)	15.6	20.7
Females, *n* (%)	9/19 (47)	10/19 (53)
Males, *n* (%)	8/14 (57)	6/14 (43)
GH treatment, *n* (%)	15/17 (88)	13/16 (81)
Fibrous dysplasia, *n* (%)	11/17 (65)	8/16 (50)
Slender bones, *n* (%)	17/17 (100)	16/16 (100)
Eating difficulties in early childhood, *n* (%)	17/17 (100)	16/16 (100)
Asthma, *n* (%)	4/17 (24)	3/16 (19)

## 4 Discussion

This study is the first comprehensive evaluation of skeletal changes in a large cohort of children and adults with MUL. Our study confirms that in addition to short stature, patients with MUL have a specific skeletal dysplasia. The characteristic features include slender and curved long bones with thick cortex and narrow medullary cavity, tall vertebral bodies, and a high prevalence of FD and fractures.

Radiographic skeletal changes were present in all 33 patients examined. The severity varied despite identical *TRIM37* genotype in all but one patient and showed no correlation with the clinical phenotype or gender. This radiographic variability is in line with the considerable variability in growth failure, facial features and other disease manifestations, as previously reported in MUL [1].

The Finnish MUL patients genotypically constitute a very homogenous group. In this study, all but one patient were homozygous for the Finnish founder mutation (Fin‐major). It is possible that the skeletal phenotype differs in subjects with other *TRIM37* mutations. However, there is no known genotype–phenotype correlation in MUL. As all published MUL‐associated *TRIM37* mutations to date seem to produce a non‐functioning protein, it is likely that the skeletal phenotype depicted in this study is not restricted only to the Finnish genotypes but is representative of all MUL subjects. Carriers of the TRIM37 gene mutation are clinically healthy and do not show any skeletal or extra‐skeletal manifestations of MUL.

FD is a significant feature of MUL as 58% of our patients displayed radiological changes typical of FD. Although histological verification of the lesions was not feasible in the present study, clinical bone biopsies of Finnish MUL patients have confirmed the diagnosis of FD. In FD, bone is replaced by disorganized fibrous tissue that may involve a single bone (monostotic FD) or multiple bones (polyostotic FD). As a result, the skeleton is weakened and prone to fractures and deformities, possibly resulting in pain and functional disability. Accordingly, also the fracture risk was clearly elevated in this study, with 52% of patients having a history of one or more fractures. All patients with polyostotic FD had sustained at least one fracture. The increased fracture risk is a new observation in MUL. The slender build of the patients presents an innate risk factor for fractures. In addition to FD, MUL patients also present with other risk factors, namely hypogonadism, feeding difficulties and malnutrition during early childhood, asthma or use of asthma medication as well as type 2 diabetes [1, 2, 3, 4, 10, 11]. Also, the short height, low muscle tone and the structural changes in the bones observed in the present study (slender diaphysis, metaphyseal overmodelling, and bowing of the long bones) may contribute to the risk of fractures in MUL. In our cohort, there was no significant difference in the characteristics between patients with or without fractures, except for a higher prevalence of FD in the fracture group. As all MUL patients develop hypergonadotropic hypogonadism as adults, and many also develop type 2 diabetes, these may be relevant additional risk factors for fractures in the adult patients. Interestingly, in this study, the prevalence of FD was higher in children than in adults. The natural course of FD lesions in patients with MUL remains unknown. Further studies are needed to evaluate whether spontaneous healing occurs.

Except for the prevalence of FD, there was no apparent difference between children and adult patients in long bone characteristics. The skeletal findings of the patients without GH treatment did not considerably differ from the others and it thus appears that the vertebral features are not secondary to GH therapy. However, conclusive significance of GH therapy on the skeletal phenotype in MUL remains unclear. Also, due to scarcity of longitudinal data, the natural course of the skeletal findings remains to be elucidated.

MUL is caused by biallelic truncating *TRIM37* mutations. The pathogenetic mechanisms are still unknown, but the function of TRIM37 as an E3 ubiquitin ligase offers an explanation for the pleiotropic features of the disorder. The ubiquitin‐proteasome system (UPS) has a fundamental role in post‐translational protein modification. The UPS regulates many pivotal cellular processes, and has an important role in cell cycle progression and cell division, signal transduction, development and cell death. The UPS is also a critical regulator of bone metabolism. Many E3 ubiquitin ligases, including some members of the TRIM protein family, such as TRIM16, TRIM21, TRIM33 and TRIM38, mediate osteogenesis or osteoclast differentiation through different pathways [12].

Recent research shows that TRIM37 has an important role in the cell cycle and regulates many centrosomal functions. Both absence and over‐expression of TRIM37 may change the levels and/or activity of key centrosomal components, leading to aberrant spindle assembly and chromosome missegregation [9]. In addition, loss of TRIM37 has been associated with increased frequency of mitotic errors [13, 14]. Interestingly, Brigant et al. showed that TRIM37 is expressed during chondrocyte mitosis and impacts their proliferation. The authors suggested that dysfunction of the chondrogenic cell cycle may lead to reduced chondrogenic cells in patients with MUL, thus leading to decreased long bone growth [15]. Furthermore, in a recent study, Brigant et al. showed that TRIM37 depletion in a Human chondrocyte cell line mainly affects the expression of extracellular matrix proteins, which could contribute to long bone abnormalities [16]. To our knowledge, the expression of TRIM37 in cells critical for normal bone formation or resorption has not been studied. The exact role of TRIM37 in bone metabolism thus remains to be elucidated.

The skeletal phenotype described in this study is distinct, but has some overlap with other dysmorphic growth disorders. Silver–Russel syndrome (SRS) is the most important differential diagnosis of MUL. SRS is genetically heterogenous and the majority of cases are sporadic. Hypomethylation of the *H19* imprinting control region (ICR) on 11p15.5 can be detected in 60% of patients and maternal uniparental disomy of chromosome 7 (mUPD7) in 5%–10% [17]. Resembling MUL, patients with SRS display severe intrauterine growth restriction without postnatal catch‐up growth, constitutional gracility and similar craniofacial dysmorphism [18]. Skeletal asymmetry with limb length discrepancy can be seen in both conditions, but is usually relatively mild in MUL. In addition, patients with moderate or severe *H19* hypomethylation may present with abnormally tall lumbar vertebrae [19].

3‐M syndrome is another primordial growth disorder with some phenotypic overlap with MUL. In addition to pre‐ and postnatal growth restriction and similar craniofacial features, patients with MUL and 3‐M also share radiological findings of slender bones and tall lumbar vertebrae. 3‐M is caused by biallelic mutations in *CUL7*, *OBSL1* or *CCDC8* [20]. Interestingly, like TRIM37, CUL7 functions as an E3 ligase and regulates the cell cycle; CUL7 has been suggested to play an important role during both mitosis and cytokinesis [21].

In conclusion, our cross‐sectional detailed radiographic evaluation of the skeletal phenotype in 33 MUL patients confirms MUL as a skeletal dysplasia with prenatal‐onset growth failure, slender bones, vertebral changes and a high prevalence of FD and fractures. MUL is not included in the most recent Nosology of genetic skeletal disorders, but our data suggest that MUL could be classified as a slender bone dysplasia (Group 21: Primordial dwarfism and slender bones group) [22]. Longitudinal studies are needed to determine the natural course of the skeletal findings and to evaluate the functional impact of skeletal manifestations. In forthcoming studies, analysis of bone quality and bone mineral density as well as biochemical markers of bone metabolism will give further insight into the bone health, individual fracture risk, and the prevalence of osteoporosis in patients with MUL. Our results indicate that more emphasis should be placed on fracture prevention. Calcium and D‐vitamin supplements should be considered in MUL patients. Furthermore, lifestyle factors supporting bone strength, such as exercise, should be advocated. Bisphosphonate treatment may be indicated for some patients. Our findings suggest an important role for TRIM37 in cellular functions governing skeletal homeostasis, including modelling and remodelling. Future studies will hopefully shed light on the pathogenetic mechanisms for the growth failure and skeletal dysplasia in MUL.

## Author Contributions

Study conception and design: Susann Karlberg, Marita Lipsanen‐Nyman, Outi Mäkitie. Collection of clinical data: Susann Karlberg, Marita Lipsanen‐Nyman. Investigation and analysis: Susann Karlberg, Sanna Toiviainen‐Salo, Marita Lipsanen‐Nyman, Outi Mäkitie. Project administration: Outi Mäkitie. Supervision: Outi Mäkitie. Writing – original draft: Susann Karlberg. Writing – review and editing: Susann Karlberg, Sanna Toiviainen‐Salo, Marita Lipsanen‐Nyman, Outi Mäkitie. All authors contributed to the article and approved the submitted version.

## Ethics Statement

This study was performed in accordance with the Declaration of Helsinki and approved by the ethical review board of Helsinki University Hospital (HUS/1190/2018 and HUS/1541/2016). All patients and/or their guardians provided an informed written consent.

## Conflicts of Interest

The authors declare no conflicts of interest.

## Data Availability

The data that support the findings of this study are available from the corresponding author upon reasonable request. The data are not publicly available due to privacy or ethical restrictions.
